# IoT Privacy Risks Revealed

**DOI:** 10.3390/e26070561

**Published:** 2024-06-29

**Authors:** Kai-Chih Chang, Haoran Niu, Brian Kim, Suzanne Barber

**Affiliations:** Department of Electrical and Computer Engineering, The University of Texas at Austin, Austin, TX 78712, USA; haoranniu@utexas.edu (H.N.); briankim31415@gmail.com (B.K.); sbarber@identity.utexas.edu (S.B.)

**Keywords:** identity, privacy, privacy policy, Internet of Things, privacy risks, entropy

## Abstract

A user’s devices such as their phone and computer are constantly bombarded by IoT devices and associated applications seeking connection to the user’s devices. These IoT devices may or may not seek explicit user consent, thus leaving the users completely unaware the IoT device is collecting, using, and/or sharing their personal data or, only marginal informed, if the user consented to the connecting IoT device but did not read the associated privacy policies. Privacy policies are intended to inform users of what personally identifiable information (PII) data will be collected about them and the policies about how those PII data will be used and shared. This paper presents novel tools and the underlying algorithms employed by the Personal Privacy Assistant app (UTCID PPA) developed by the University of Texas at Austin Center for Identity to inform users of IoT devices seeking to connect to their devices and to notify those users of potential privacy risks posed by the respective IoT device. The assessment of these privacy risks must deal with the uncertainty associated with sharing the user’s personal data. If privacy risk (R) equals the consequences (C) of an incident (i.e., personal data exposure) multiplied by the probability (P) of those consequences occurring (C × P), then efforts to control risks must seek to reduce the possible consequences of an incident as well as reduce the uncertainty of the incident and its consequences occurring. This research classifies risk according to two parameters: expected value of the incident’s consequences and uncertainty (entropy) of those consequences. This research calculates the entropy of the privacy incident consequences by evaluating: (1) the data sharing policies governing the IoT resource and (2) the type of personal data exposed. The data sharing policies of an IoT resource are scored by the UTCID PrivacyCheck^™^, which uses machine learning to read and score the IoT resource privacy policies against metrics set forth by best practices and international regulations. The UTCID Identity Ecosystem uses empirical identity theft and fraud cases to assess the entropy of privacy incident consequences involving a specific type of personal data, such as name, address, Social Security number, fingerprint, and user location. By understanding the entropy of a privacy incident posed by a given IoT resource seeking to connect to a user’s device, UTCID PPA offers actionable recommendations enhancing the user’s control over IoT connections, interactions, their personal data, and, ultimately, user-centric privacy control.

## 1. Introduction

The proliferation of Internet of Things (IoT) devices has brought convenience and connectivity into our daily lives, yet it has also raised significant concerns regarding user privacy. Despite the increasing reliance on IoT technology, users often remain uninformed about the privacy risks associated with the devices and applications they interact with. This lack of awareness stems from several factors.

Firstly, privacy policies associated with IoT devices and applications, which outline how user data are collected, used, shared, and monetized, are often lengthy and complex, leading to low engagement from users. Research indicates that a mere fraction of users actually read privacy policies, with only around 4.5% claiming to consistently do so [1]. Moreover, website observations suggest that the proportion of users reviewing privacy policies may be even lower, hovering around 1%. The readability of these policies presents a significant barrier, requiring a level of comprehension typically associated with college education.

Secondly, users frequently lack visibility into the connections established by IoT devices and applications. They are often unaware of the devices and applications accessing their personal data and the objectives they are being utilized for. This absence of transparency impairs the ability of users to make informed choices concerning their privacy.

In response to these challenges, this research proposes a novel approach to empower users with unprecedented knowledge and control over those IoT devices and applications connecting to their devices. The approach leverages the capabilities of the Personalized Privacy Assistant (PPA) in conjunction with the PrivacyCheck^™^ tool and UTCID IoT Identity Ecosystem, all developed by the University of Texas Center for Identity (UTCID).

The UTCID PPA serves to deliver user-centric privacy control by detecting IoT connections with IoT privacy infrastructures, providing users with real-time insights into the IoT resources (devices, services, and apps) accessing their data and notifying those users of potential privacy risks posed by the respective IoT resource. The assessment of these privacy risks must deal with the uncertainty associated with sharing the user’s personal data. If privacy risk (R) equals the consequences (C) of an incident (i.e., personal data exposure) multiplied by the probability (P) of those consequences occurring (C × P), then efforts to control risks must seek to reduce the possible consequences of an incident as well as reduce the uncertainty of the incident and its consequences occurring. This research classifies risk according to two parameters: expected value of the incident consequences and uncertainty (entropy) of those consequences. This research calculates the entropy of the privacy incident consequences by evaluating: (1) the data sharing policies governing the IoT resource and (2) the type of personal data exposed. The data sharing policies of an IoT resource are scored by the UTCID PrivacyCheck^™^ using machine learning to “read” and score the IoT resource privacy policies against metrics set forth by best practices and international regulations. The UTCID IoT Identity Ecosystem uses empirical identity theft and fraud cases to assess the entropy of privacy incident consequences involving a specific type of personal data, such as name, address, Social Security number, fingerprint, and user location. By understanding the entropy of a privacy incident posed by a given IoT resource seeking to connect to a user’s device, UTCID PPA offers actionable recommendations enhancing the user’s control over IoT connections and the exposure of their personal data.

Concurrently, UTCID PrivacyCheck^™^ evaluates the privacy policies of the organizations associated with these IoT resources, enabling users to comprehend the privacy implications of their interactions. By leveraging these insights of connecting IoT devices and applications as well as the associated privacy risks, the framework offers personalized recommendations to users, thereby enhancing their ability to manage privacy risks effectively.

By bridging the gap between user awareness and IoT device connectivity, this research aims to address the pressing need for user-centric privacy management in the IoT ecosystem.

The following are the primary contributions of this paper:

- **Empowering users with enhanced privacy awareness**: By integrating the Personalized Privacy Assistant (UTCID PPA) and UTCID PrivacyCheck^™^, this research offers users unprecedented insight into the connections established by IoT devices and the associated entropy of privacy incident consequences and resulting risks. This heightened awareness empowers users to make informed decisions about their privacy, thereby promoting digital autonomy, agency, and self-determination.
- **Streamlining privacy management processes**: The proposed framework facilitates the seamless detection of IoT connections and automated evaluation of privacy policies, simplifying the otherwise cumbersome task of privacy management. By offering personalized recommendations based on real-time assessments, users can efficiently reduce entropy of privacy incident consequences, mitigate privacy risks, and maintain control over their personal data across diverse IoT interactions.
- **Contributing to the evolution of privacy-enhancing technologies**: Through its innovative integration of machine learning and the privacy policy analysis tool PrivacyCheck^™^, as well as the empirical study and computation modeling of personal data values and risk exposures in the UTCID IoT Identity Ecosystem, this research advances the frontier of privacy-enhancing technologies. By addressing critical challenges such as privacy policy transparency, user awareness, and the privacy risks associated with the exposure of specific personal data, the proposed UTCID PPA sets a precedent for future developments in user-centric privacy management solutions, driving progress towards a more privacy-respecting digital landscape.

The structure of this paper is as follows. Section 2 provides a concise overview of related research. Section 3 introduces the proposed solution, discussing various problem statements and details of system implementation. Section 4 elaborates on the experimental results for UTCID PPA and UTCID PrivacyCheck^™^ and includes comparisons with existing work along with suggestions for future research. Section 5 summarizes the conclusions of the study.

## 2. Related Work

In recent years, privacy-enhancing technologies (PETs) have emerged as crucial tools that enable greater control over a user’s online privacy. Among these technologies, efforts to summarize and visualize complex online privacy policies have gained significant traction. In this section, we review existing work that relates to this specific aspect.

### 2.1. Devices and Privacy

Related work concerning devices and personal privacy can be categorized into three main areas: surveys, research tools, and user empowerment tools. Surveys such as the one conducted by Reardon et al. [2] reveal the extensive unauthorized access prevalent in mobile apps. This unauthorized access is not limited to commonplace data elements such as names or emails but extends to more sensitive and invasive data elements, including geolocation and personal health information [3]. The situation is compounded by the discovery of numerous third-party Android libraries that are collecting and subsequently leaking data [4]. This poses a significant privacy risk to users who are typically unaware and ill-equipped to detect or counteract such breaches apart from ceasing app usage.

In response, many research tools have been developed to facilitate further investigations in this field. For instance, Alazab et al. [5] developed a robust classification model that detects malware mobile apps. It accomplishes this by categorizing an app’s high-profile API calls into three levels of risk—ambiguous, risky, and disruptive—in increasing order of severity. This classification reflects the frequency and nature of the API requests in benign versus harmful applications, enabling the model to accurately assess the likelihood of an app being malware.

Furthermore, previous work in user empowerment tools, often in the form of a mobile app, aim to enhance accessibility and user control. A notable example is ALManager, introduced by Liu et al. [6]. This Android app gives users the ability to manage any information gathered by analytics libraries embedded in other apps on the user’s phone. ALManager operates by first displaying to users the information being collected by these libraries. Based on this visibility, users can then decide which apps may continue to collect data and which should be restricted from further data collection.

### 2.2. Privacy Risk Assessment

Research addressing privacy risks of mobile applications can be divided into three categories: the mobile app’s permission analysis, the mobile app’s privacy policy analysis, and mobile security and privacy framework.

Numerous studies have shown that mobile applications often request excessive permissions for a user’s identity attributes. For instance, a survey [7] examined the issue of over-declared permissions by operating systems across various types of popular mobile phones. Additionally, an Android API tool [8] was developed to tackle the problem of permission over-declaration.

Furthermore, existing research addresses the mitigation of digital privacy risks. Zaeem et al. [9,10,11] introduced a method of analyzing privacy policies and generating an automated summary. A set of metrics [12] was developed by formulating a series of questions, reviewing privacy policies in the context of those questions, and using the corresponding answers to those questions for a respective privacy policy to score privacy risks posed by the respective privacy policies. Polisis, a framework proposed by Harkous et al. [13], utilized neural network classifiers to perform analysis on such privacy policies.

The impact of stolen identity attributes goes beyond just the theft of information; it can also result in financial and emotional loss for the user. In an effort to better understand the scope and origin of such identity breaches, a tool [14] was designed to pinpoint any potential vulnerabilities in mobile apps by analyzing how cloud APIs are utilized.

### 2.3. Privacy Assistant

IoT users frequently engage with unfamiliar technologies. This lack of knowledge, combined with insufficient means to effectively manage myriad notifications and settings, poses challenges for users of IoT resources. Moreover, users often do not realize which devices, apps, or services in their vicinity are collecting their information, nor do they understand the personal consequences of such data collection.

Das et al. [15] created a privacy assistant that uses machine learning techniques to remember a user’s privacy expectations and preferences. This assistant can discern whether the collection of identity attributes is significant enough to notify the user and only communicates information that the user is likely to value. Feng et al. [16] developed a framework that assists developers in creating meaningful privacy options within their systems or applications. Their research also demonstrated how this framework could be applied to an IoT privacy assistant. Ayci et al. [17] designed a privacy assistant powered by deep learning that adapts its recommendations to the user’s preferences and assesses the certainty of its recommendations. If uncertain, it queries the user, allowing the assistant to personalize its advice further. Stöver et al. [18] performed a series of user studies to define their own implementation of a privacy assistant and to understand how to design it effectively. Their survey investigated the functionalities that the privacy assistant should have and identified which user groups were most appropriate for different functions.

### 2.4. Summary

Table 1 provides a comprehensive summary of the related work categories discussed above.

Existing research typically analyzes one mobile app at a time to detect the sharing and generation of identity attributes. This approach fails to provide a thorough analysis of the collection, sharing, and abuse of identity attributes. It also lacks a detailed examination of which data qualify as identity attributes, the interdependencies between identity attributes, the value of these attributes, and the risks associated with sharing or abusing personally identifiable information (PII). In contrast, the research presented in this paper develops a comprehensive model that encompasses identity attributes, attribute dependencies, attribute values, and associated risks, based on empirical data collected from extensive testing of over 200 mobile apps [19,20].

Existing related work detects actual data transmission and therefore can only allow for reactive solutions to describe the resulting consequences of personal data sharing. This research embodied in the UTCID PPA offers a preventive risk assessment solution by preemptively evaluating privacy vulnerabilities and alerting users to potential repercussions of mobile app data collection and shared identity attributes.

The research discussed in this paper develops a personal privacy assistant that is designed to inform users about the potential consequences of connecting to IoT resources (devices, apps, and services) and sharing of a user’s PII (identity attributes), thereby giving users awareness and agency to prevent those risks before consequences are experienced.

## 3. UTCID Personalized Privacy Assistant with UTCID PrivacyCheck^™^ Risk Assessment

The UTCID Personalized Privacy Assistant (UTCID PPA) is a mobile application designed to assist users in identifying nearby IoT resources (devices, apps, and services). Through the use of IoT Resource Registries (IRR) data, the UTCID PPA determines which devices and services in the vicinity of the user’s current location are capable of collecting personal information. By accessing the IRR, the UTCID PPA provides users with a list of registered devices along with details such as functionality, ownership, and privacy practices. Users are also presented with options to either allow or disallow the collection and provision of PII by these IoT resources (devices, apps, or services).

To assist users in making decisions about the risks they may incur as a consequence of agreeing to connect with an IoT resource and agreeing to the collection, use, and sharing of their PII by a respective IoT resource, the UTCID PPA leverages the UTCID IoT Identity Ecosystem [19,21] to assess a user’s privacy risks (Section 3.3). The UTCID PPA informs users about IoT resources collecting their PII, evaluates the privacy risks associated with the specific PII (identity attributes) to be collected using empirical risk data on every identity attribute found in the UTCID Identity Ecosystem, and then summarizes the overall risk to the user using the UTCID PPA Privacy Risk Score Calculation Algorithm. This algorithm assesses the impact of data collection on user privacy by considering the risks associated with collecting, using, and sharing identity attributes collected by respective IoT resources.

In addition to giving users an analysis of the inherent risks in sharing specific PII (identity attributes) as described above, the UTCID PPA analyzes the privacy policies of an identified IoT device manufacturer or application vendor to better understand the respective organization’s data collection, use, and sharing policies. The UTCID PPA IoT Device Analysis Feature (Section 3.3) integrates with UTCID PrivacyCheck^™^ to find the relevant privacy policy and score the organization’s privacy policy according to known U.S. and international regulations.

**Figure 1:**
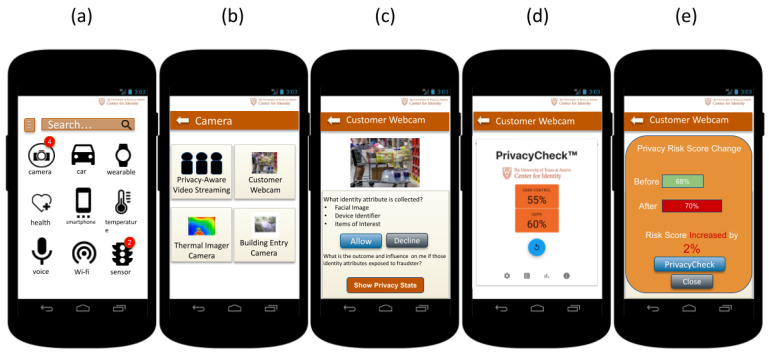
The graphic user interface for UTCID Personalized Privacy Assistant (UTCID PPA) displays a list of available devices and services (**a**,**b**), as well as detailed information about data collection and usage practices of certain resources (**c**). It also includes privacy risk analysis for the user (**d**,**e**).

Figure 1 illustrates a sample graphical user interface of the UTCID PPA. In a scenario where a user enters a store equipped with a smart camera system utilizing facial recognition and behavioral tracking, the UTCID PPA notifies the user of the presence of such devices. In Figure 1a, UTCID PPA notifies the user about the presence of such devices with the red icon in the top right of each image of different types of devices (e.g., camera, car, wearable, smartphone, etc.). By clicking the corresponding image, the UTCID PPA shows different devices of the same type nearby, as is shown in Figure 1b. By tapping the “Customer Webcam”, the UTCID PPA provides information about the Customer Webcam data collection, use, and sharing practices, which is shown in Figure 1c. Upon assessing the associated privacy risks and consulting UTCID PrivacyCheck^™^ for additional insights, which is shown in Figure 1d,e, the user can make informed decisions about whether to accept and allow the Customer Webcam to connect to their device and thereby accept the data collection and sharing practices of the Customer Webcam smart camera system.

**Figure 2:**
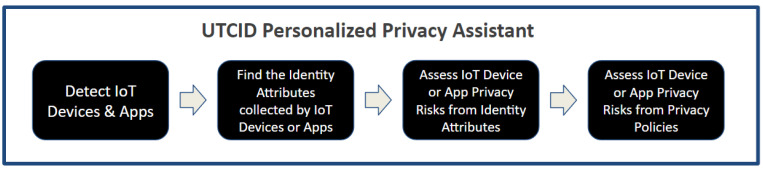
Operational capabilities of the UTCID Personalized Privacy Assistant.

Figure 2 highlights the operational capabilities of the UTCID Personal Privacy Assistant (UTCID PPA), giving the user awareness and empowering the user to make informed decisions about IoT resources (devices, apps, and services) seeking connections to their personal devices. In other words, UTCID PPA gives users real insights about privacy risks posed by respective IoT resources, helping users decide whether to allow or prevent that IoT connection. Figure 2 outlines the computational process UTCID PPA uses to: (1) detect IoT resources (devices, applications, and services) seeking to connect to the user’s device; (2) assess the privacy risks associated with those IoT resources, then (3) inform the user of any privacy concerns linked with exposing the respective PII data (identity attributes) the respective IoT resource seeks to collect; and (4) assess the risks posed by the privacy policies of the respective IoT device manufacturer, IoT app vendor, or IoT service provider.

The following sections describe in detail the UTCID PPA capabilities highlighted in Figure 2.

### 3.1. UTCID PPA Detects IoT Devices and Apps

**Figure 3:**
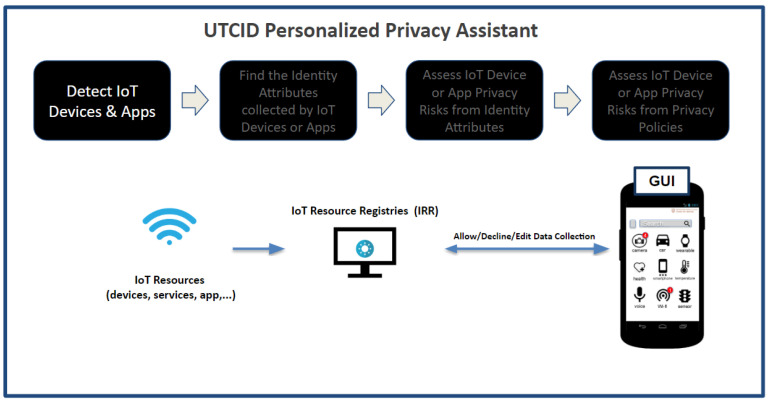
The UTCID Personalized Privacy Assistant detects nearby IoT devices and apps by accessing the IRR.

The UTCID PPA facilitates registration for IoT resources (e.g., devices, services, apps), allowing them to provide their location and disclose the PII they collect. Upon connecting a mobile phone to the UTCID PPA privacy infrastructure, users can identify nearby IoT resources that may collect their PII (shown in Figure 3). The infrastructure utilizes the user’s current GPS location to determine such resources, including various smart entities such as houses, cameras, and applications, as well as services like location tracking and body temperature analysis. The UTCID PPA interacts with this infrastructure (IRR), analyzing data provided about the collected identity attributes [15,16,22]. Recognizing that users can only control what they are aware of, this connection serves as a critical initial step by the UTCID PPA to empower users to manage their privacy within the IoT.

### 3.2. UTCID PPA Finds the Identity Attributes Collected by IoT Devices or Apps

**Figure 4:**
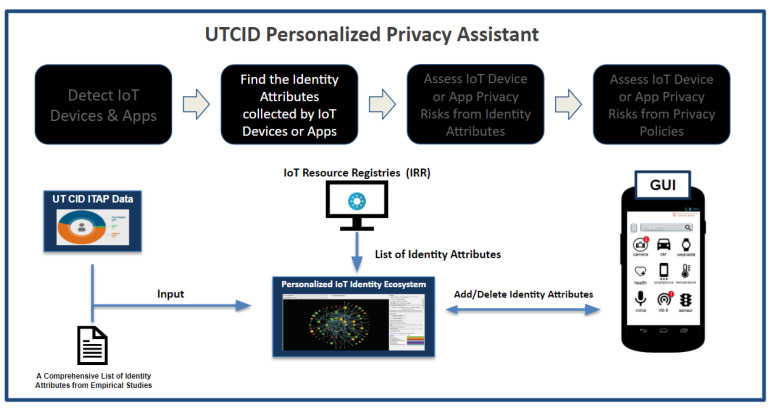
The UTCID Personalized Privacy Assistant finds the identity attributes collected by IoT devices or apps in the UTCID IoT Identity Ecosystem.

Figure 4 displays the process by which the UTCID PPA records and assesses PII, also referred to as identity attributes, collected by a respective IoT device seeking connection to the user’s device. Identity attributes constitute a very important and relevant term to use in this research, as the PII to be analyzed truly is a list of attributes identifying individuals from a list of:

- What users KNOW (e.g., name, address, mother’s maiden name),
- What users HAVE (e.g., assigned credit card, SSN card),
- What users ARE (e.g., physical biometrics such as fingerprint or photographs), and
- What users DO (e.g., patterns of life such as geolocation patterns, website visit patterns, shopping patterns).

The UTCID PPA is instrumental in the assessment and management of the identity attributes gathered from IoT devices. In addition to leveraging empirical studies of the types of identity attributes collected by IoT resources [19], the UTCID PPA leverages the comprehensive data housed within the IRR to determine the spectrum of PII (identity attributes) collected by each IoT resource. This strategic approach ensures accuracy and efficiency in identifying relevant identity attributes.

Once armed with the list of identity attributes collected from an IoT resource, in order to give users some insights about potential harms and privacy risks, the UTCID PPA utilizes this information to calculate a privacy risk score before and after connecting with the respective IoT resource. This assessment is facilitated by the UTCID IoT Identity Ecosystem, a structured framework that categorizes and organizes identity attributes into descriptors for both individuals and devices. By leveraging the UTCID IoT Identity Ecosystem, the UTCID PPA can accurately gauge the impact of collected identity attributes on privacy risk, empowering users with valuable insights into their data exposure.

The UTCID IoT Identity Ecosystem is a structured graph-based representation of PII, identity attributes, for both personal attributes (e.g., blood type, postal address, name) and device attributes (e.g., model number, name, GPS location). It is noteworthy that certain attributes may pertain to both individuals and devices, such as GPS location, highlighting the intricate relationship between human users and technological devices.

**Figure 5:**
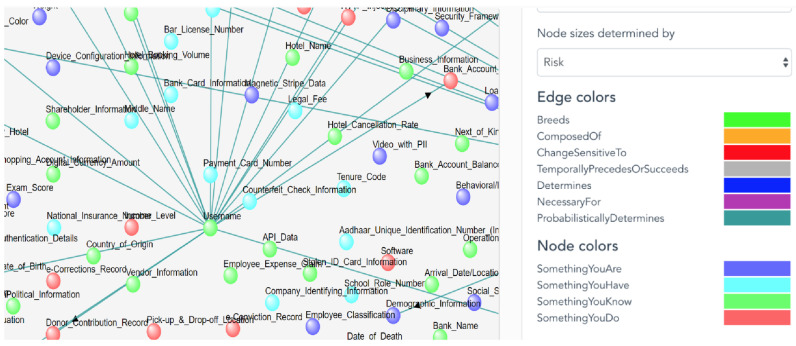
A snapshot of the UTCID IoT Identity Ecosystem showing graph representation of the identity attributes, ways to select the size and color of identity attribute nodes, and the relationships/dependencies (edge between graph nodes).

Figure 5 illustrates the interconnectedness of these identity attributes within the IoT ecosystem. In this figure, the size of each node within the diagram is determined by the risk of exposure as set by the filter on the right, with different colors employed to differentiate the types of identity attributes. Each node is labeled with the name of its respective identity attribute. Table 2 provides concrete examples of identity attributes attributed to IoT resources, demonstrating the breadth and complexity of data collected by these resources [23,24,25].

Data in the UTCID Identity Ecosystem, including the identity attributes and the identity attribute values, identity attribute exposure rates/frequency, negative consequences of identity attribute exposure, and dependencies between identity attributes, was formulated based on empirical data from the UTCID Identity Threat Assessment and Prediction (ITAP) [26,27] research project. The UTCID ITAP models and analyzes identity theft and fraud cases, including the vulnerabilities that lead to data compromises and breaches, as well as the consequences of these data exposures. The project focuses on identifying the types of identity attributes that are exposed, how frequently each attribute is compromised, and the ramifications of these exposures, which can include financial loss, reputation damage, and emotional distress for the victims. Over 6000 incidents have been modeled [28]. By examining these identity theft, fraud, and abuse cases, UTCID ITAP has compiled a list of reported identity attributes. Each attribute is characterized by several factors, such as its monetized value, risk (probability) of exposure, verification accuracy, and other characteristics derived from the data.

The risk (i.e., probability) of exposure for any identity attribute *A* is estimated using the following equation:

(1) $$risk\left(A\right)=\frac{\#cases(A)}{\#cases},$$

where #cases(A) is the number of theft cases in which this identity attribute was exposed or misused by a criminal, and #cases is the total number of theft cases in the ITAP. The monetized value of any identity attribute *A* is defined as the average dollar value lost in cases where attribute *A* was misused by identity criminals. This is described by the following equation:

(2) $$value\left(A\right)=\frac{\sum_{c\in cases(A)}V_{c}}{\#cases(A)},$$

where $V_{c}$ is the dollar value loss incurred in a given case. For identity attributes *A* and *B*, the probability of the edge between them is calculated as follows:

(3) $$E_{AB}=P\left(Breach\left(B\right)|Breach\left(A\right)\right).$$

This equation indicates that, given all cases involving attribute *A*, the rate at which attribute *B* is also involved is used to determine the conditional probability of the edge connecting *A* and *B*.

So far, ITAP has discovered over 600 identity attributes related to these incidents. These attributes form the foundational reference data for populating the UTCID Identity Ecosystem, where each attribute is represented as a node in the graph-based structure of the ecosystem. Through collaboration with experts and in-depth studies, this research systematically populated identity attributes of devices from the UTCID Identity Ecosystem, categorized these attributes, and established any connections among the attributes describing IoT resources and those pertaining to individuals as defined in prior research endeavors [21].

### 3.3. UTCID PPA Assesses IoT Device or App Privacy Risks from Identity Attributes

To conduct the privacy risk score analysis, it is essential to first define the concepts of privacy risk within the context of the UTCID IoT Identity Ecosystem. Privacy risk is defined as the likelihood (probability) of incurring monetary loss (privacy incident consequences) due to the exposure, theft, or misuse (privacy incident) of personally identifiable information (identity attributes or personal data). A higher privacy risk score suggests the higher probability of a privacy incident or higher probability of greater potential financial loss that an individual could encounter if their identity attributes are exposed, stolen, or misused. Moreover, entropy can be used to represent uncertainty (probability) as part of the privacy risk assessment model, namely, the entropy of the privacy incident occurrence exposing a specific type of personal data such as SSN, name, email, fingerprint, or location, and the entropy of the privacy incident consequences resulting in monetary loss. In the context of the UTCID IoT Identity Ecosystem, this could mean a wider range of potential threats or vulnerabilities that could lead to exposure.

The UTCID PPA framework integrates entropy into its calculation of privacy risk scores through two dynamic properties that relate to the probability of exposure (privacy incident) and probability of consequence, namely: *Accessibility* and *Post Effect*. *Accessibility* refers to how an identity attribute’s ancestors within the UTCID IoT Identity Ecosystem can influence its probability of being exposed. This factor allows for the modification of the original exposure probability of an identity attribute by superimposing the effects of its connectivity within the ecosystem. *Post Effect*, on the other hand, deals with the escalation in the probability of negative consequences, particularly property loss, following an exposure event. This can then be superimposed on the original property risks or losses associated with the identity attribute.

**Figure 6:**
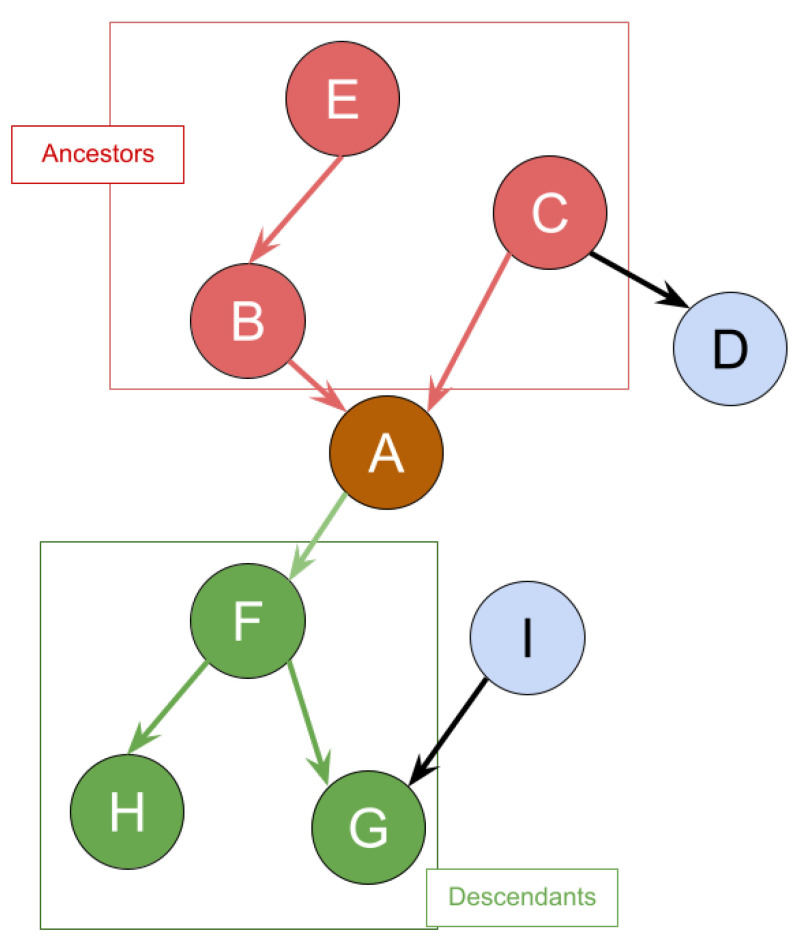
An example of ancestors and descendants.

In the context of the UTCID IoT Identity Ecosystem, as described by the graph in Figure 6, identity attribute *A* has ancestor attributes B,C, and *E*. This implies that there exists a direct or indirect path from each of these ancestor attributes to *A*. Identity attribute *D* does not share such a path and therefore is not considered an ancestor of *A*. To analyze the risk associated with the exposure of identity attribute *A* after any of its ancestors ($A_{k}$) are exposed, we can utilize Bayesian inference to update the probability of exposure for *A* based on the exposure of its ancestors. Let Anc(A) represent the set of all ancestors of *A*. If an ancestor $A_{k}$ (where $A_{k}\in Anc\left(A\right)$) is exposed, the posterior probability of *A* being exposed is denoted as $P^{\prime }\left(A\right)$. This updated probability, $P^{\prime }{\left(A\right)}_{A_{k}}$, reflects the likelihood that *A* will be exposed following the exposure of $A_{k}$. To compute the percentage increase in the probability of the exposure of *A* following the exposure of $A_{k}$, the following formula can be used: $P^{\prime }{\left(A\right)}_{A_{k}}-P\left(A\right)$. Based on this formula, the total sum of the percentage increase in the probability of the exposure of *A* can be given as follows:

(4) $$AC\left(A\right)=\sum_{A_{k}}\left(P^{\prime }{\left(A\right)}_{A_{k}}-P\left(A\right)\right),$$

where $A_{k}\in Anc\left(A\right)$, and AC(A) represents the *Accessibility* of *A*.

In this work, the new risk (real risk) of exposure for identity attribute *A* is defined as follows:

(5) $$P^{\prime }\left(A\right)=P\left(A\right)+AC\left(A\right),$$

where the aforementioned *Accessibility* of *A* is again represented as AC(A).

In the scenario where identity attribute *A* in Figure 6 is breached, the exposure of its descendants F,G, and *H* is also likely to be affected due to the interconnected nature of the identity attributes. Using Bayesian inference, we can determine how the breach of *A* impacts the probability of exposure for each descendant in the set Des(A), where Des(A) is the set of all descendants of *A*. If we denote each descendant of *A* as $A_{k}$, then the posterior probability of exposure for $A_{k}$ following the breach of *A* is $P^{\prime }\left(A_{k}\right)$. The difference in the probability of exposure due to the breach can be quantified as $P^{\prime }\left(A_{k}\right)-P\left(A_{k}\right)$. Furthermore, each identity attribute *A* is associated with a liability value L(A), which quantifies the potential monetary loss associated with the breach of that attribute. Thus, the increase in potential monetary loss for a descendant identity attribute $A_{k}$ due to the increased probability of exposure can be calculated as $L\left(A_{k}\right)\times \left(P^{\prime }\left(A_{k}\right)-P\left(A_{k}\right)\right)$.

As a result, the total monetary loss increase of the descendant set Des(A) can be shown as follows:

(6) $$PE\left(A\right)=\sum_{A_{k}}L\left(A_{k}\right)\times \left(P^{\prime }\left(A_{k}\right)-P\left(A_{k}\right)\right),$$

where $A_{k}\in Des\left(A\right)$, and we call PE(A) the *Post Effect* of *A*.

In this work, the new loss value (real value) for identity attribute *A* is defined as follows:

(7) $$L^{\prime }\left(A\right)=L\left(A\right)+PE\left(A\right),$$

where PE(A) denotes the *Post Effect* of *A*.

To assess the impact of identity attribute exposure, the expected loss of an identity attribute *A* can be defined as follows:

(8) $$Exp\left(A\right)=P^{\prime }\left(A\right)\times L^{\prime }\left(A\right).$$

Given that the expected loss values across the ecosystem range from 0 to $10^{7}$, this range is too broad for effective data analysis and comparison. To address this, the natural logarithm of each expected loss is taken: ln(Exp(A)). This transformation compresses the scale of loss values, making them more manageable and easier to compare. Furthermore, to reflect the increased risk and potential losses linearly, the highest expected loss in the ecosystem is identified, and its natural log is taken. The privacy risk score for an identity attribute *A* is then defined as follows:

(9) $$score_{risk}\left(A\right)=\frac{ln(Exp(A))}{Max},$$

where Max is the maximum logged value of expected loss across all identity attributes. This formula normalizes the risk score to a value between 0 and 1, allowing for consistent and relative comparisons across different identity attributes.

**Figure 7:**
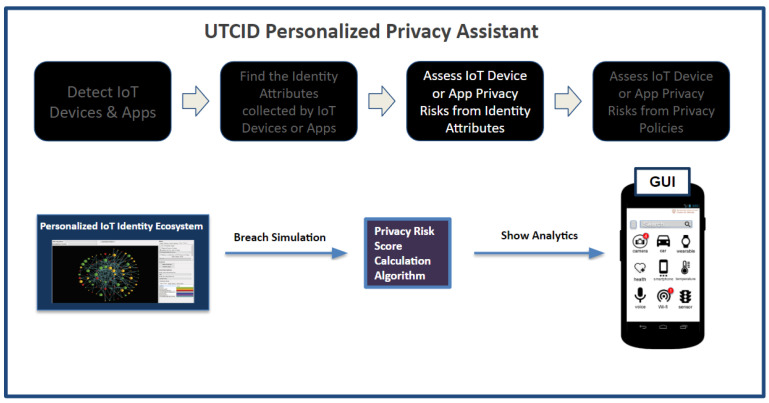
The UTCID Personalized Privacy Assistant calculates the privacy risk resulting from specific identity attributes collected by IoT devices or apps by leveraging the breach simulation from the UTCID IoT Identity Ecosystem.

When considering an IoT resource *S* that collects multiple identity attributes, say *N* of them, the set of identity attributes collected can be represented as $ID_{S}={\left\{A_{i}\right\}}_{i=1:N}$. The total privacy risk score for the dataset collected by *S* can be calculated as follows:

(10) $$Score\left(S\right)=\frac{1}{Total}\sum_{i=1}^{N}score_{risk}\left(A_{i}\right),$$

where Total represents the sum of all risk scores within the UTCID Identity Ecosystem, ensuring that the aggregate privacy risk score for *S* is also normalized between 0 and 1. This comprehensive scoring mechanism is visualized in Figure 7, with detailed mathematical symbols summarized in Table 3.

Entropy in the UTCID IoT Identity Ecosystem represents the degree of uncertainty and unpredictability associated with the exposure of specific identity attributes. It is a measure of the diversity and variability of potential vulnerabilities, threat incidents, and consequences within the ecosystem. High entropy indicates a greater range of possible outcomes and a higher level of uncertainty, which in turn suggests a more complex and less predictable risk landscape.

In the context of privacy risk assessment, entropy helps to quantify the uncertainty regarding the exposure (privacy incidents) probabilities of different identity attributes. This quantification is crucial for understanding the potential risks and for designing effective risk mitigation strategies. By integrating entropy into the privacy risk assessment model, the UTCID PPA can better account for the unpredictability and variability of identity-related risks. Higher entropy values signal that there are many different ways in which identity attributes can be exposed, reflecting a wider array of potential threat incidents and consequences. This insight is invaluable for creating a robust risk assessment framework that can adapt to the dynamic and evolving nature of the IoT ecosystem.

To demonstrate the effectiveness of the UTCID PPA, prior research [20,29] evaluated the privacy risks associated with the 10 most popular open-source Android apps. The results of this evaluation are displayed in Table 4. In this context, higher privacy scores signify that an app represents a greater risk to user privacy.

Consider the app Telegram. To evaluate the privacy risks associated with Telegram, the UTCID PPA follows a structured analytical process. Initially, it begins by identifying the specific identity attributes associated with Telegram that it collects and shares. This is done by accessing a list of identity attributes from the IRR. Following the identification process, the UTCID PPA moves to the risk and liability assessment stage. Here, it consults the UTCID IoT Identity Ecosystem to ascertain the risk of exposure (e.g., P(A) for identity attribute *A*) and the liability value (e.g., L(A) for identity attribute AL) for each of these identity attributes. This information forms the basis for the next steps. The third step involves applying Formulas (4)–(7) for *Accessibility* and *Post Effect* to each identity attribute collected by Telegram. Once these adjustments are made, the UTCID PPA then utilizes the refined data to calculate the individual privacy risk scores for each identity attribute associated with Telegram. This is achieved through the application of Formula (8). Finally, the overall privacy risk presented by Telegram is determined by aggregating these individual scores using Formula (9). For Telegram, this comprehensive analysis results in a privacy risk score of 73.99, as displayed in Table 4. Overall, the integration of entropy into the privacy risk assessment process enhances the understanding of uncertainty and diversity within the UTCID IoT Identity Ecosystem, enabling more effective risk management and protection of an individual’s privacy.

### 3.4. UTCID PPA Assesses IoT Device or App Privacy Risks from Privacy Policies

**Figure 8:**
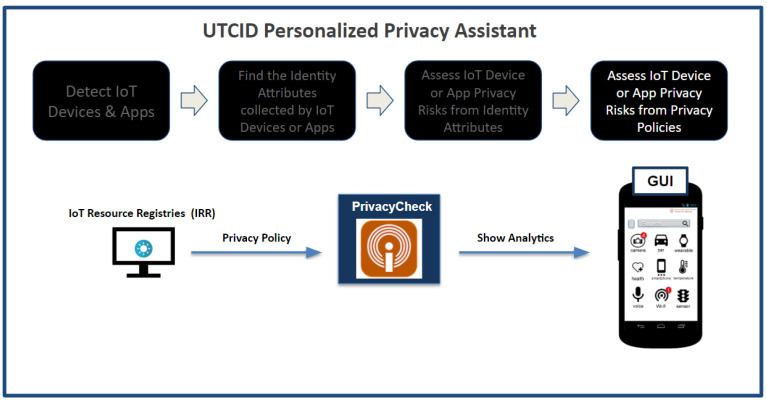
The UTCID Personalized Privacy Assistant assesses the UTCID PrivacyCheck^™^ to acquire more insights for users.

From the IRR, the UTCID PPA is able to access the privacy policy of the IoT resource, which is the step shown in Figure 8. UTCID PPA uses the UTCID PrivacyCheck^™^ to enhance a user’s comprehension of an IoT resource’s data sharing policies described in its privacy policy and to calculate the uncertainty (entropy) of privacy incident consequences resulting from the privacy policy of a respective IoT resource seeking to connect to the user’s device. This research classifies risk according to two parameters: expected value of the incident consequences and uncertainty (entropy) of those consequences. As part of the entropy calculation of the privacy incident consequences, UTCID PPA leverages PrivacyCheck^™^ to evaluate data sharing policies governing the IoT resource. The data sharing policies of an IoT resource are scored by the UTCID PrivacyCheck^™^ using trained deep learning to “read” and score the IoT resource privacy policies against metrics set forth by best practices and international regulations. Specifically, the UTCID PrivacyCheck^™^ is a machine learning-based privacy enhancing technology (PET) that evaluates a respective online privacy policy describing how an organization intends to collect, use, and share user data (i.e., PII or identity attributes) [11,30].

The UTCID PPA searches for the privacy policy of the IoT resource manufacturer then gives its URL to UTCID PrivacyCheck^™^. The UTCID PrivacyCheck^™^ takes a privacy policy as an input and answers 20 questions, giving users easy-to-understand stoplight scores (green, yellow, and red), as shown in Table 5. The UTCID PrivacyCheck^™^ answers questions rooted in the Organization for Economic Cooperation and Development [30] and Federal Trade Commission FIPPs (Fair Information Practice Principles) [31] and GDPR (European General Data Protection Regulation) [11,32]. Prior research describes the process in which UTCID PrivacyCheck^™^ questions were selected and how LightGBM machine learning models were trained and deployed to score privacy policies according to these 20 questions [33]. Furthermore, UTCID PrivacyCheck^™^ has been successfully applied across various applications. For instance, it has been employed to investigate the impact of GDPR on the landscape of privacy policies [11], to compare privacy policies between the public and private sectors [34], to analyze privacy policies across different industries [10], and to study patterns in the usage of PETs [35].

**Figure 9:**
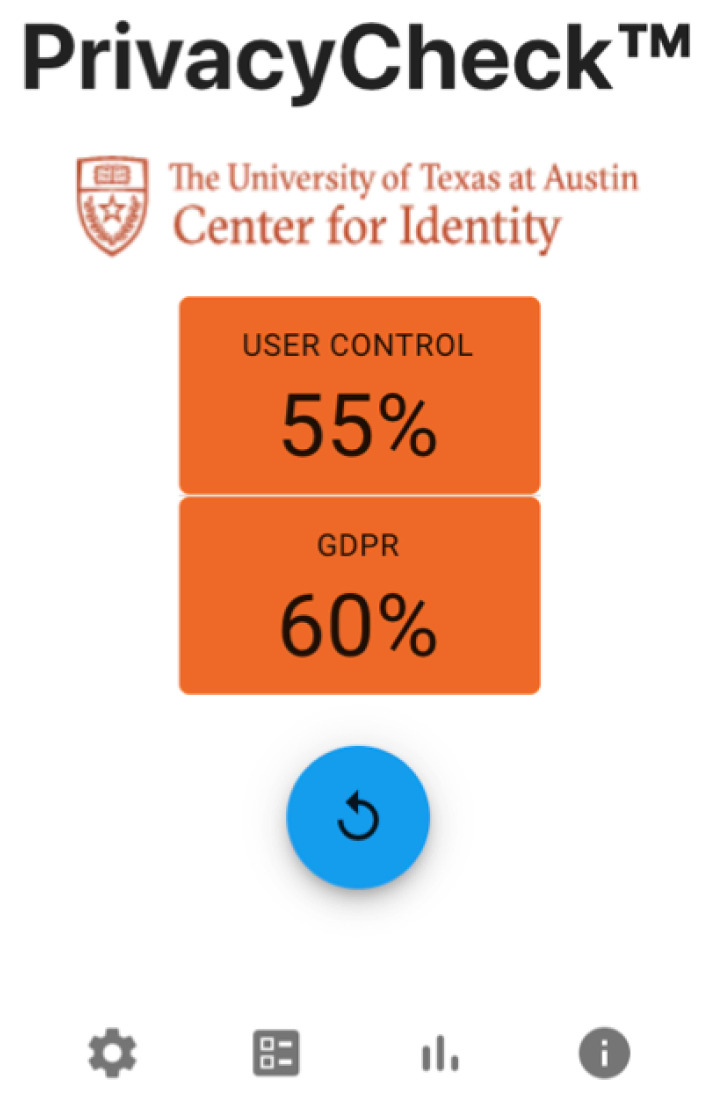
UTCID PrivacyCheck^™^ run panel: User Control and GDPR scores for a sample privacy policy.

**Figure 10:**
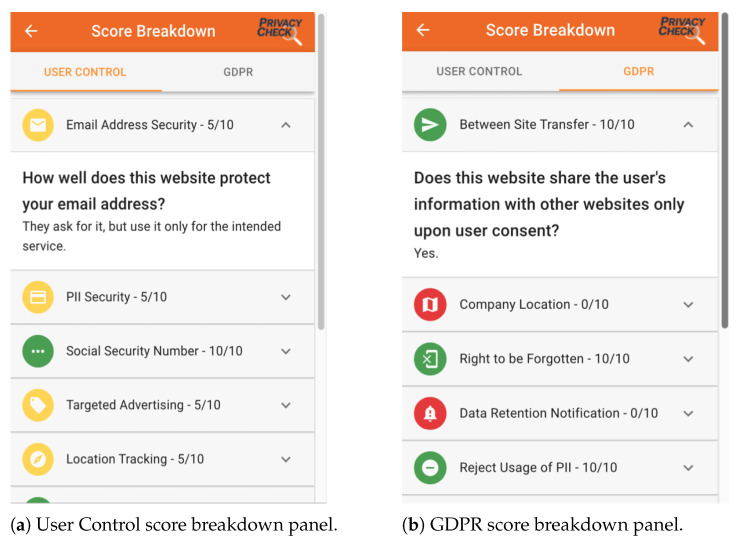
UTCID PrivacyCheck^™^ breakdown panel.

Figure 9 shows the UTCID PrivacyCheck^™^ display showing the compliance of the respective privacy policy with the U.S. and international regulations associated with these 20 questions. The User Control score is given based on how well the website performs based on the 10 User Control questions. Likewise, the GDPR score is based on the privacy policy’s adherence with the 10 GDPR questions. As seen in Figure 9, two overall scores represent the average of the compliance score for each respective User Control and GDPR question. For both overall scores, users have the ability to check the compliance scores for each question shown in Figure 10.

**Figure 11:**
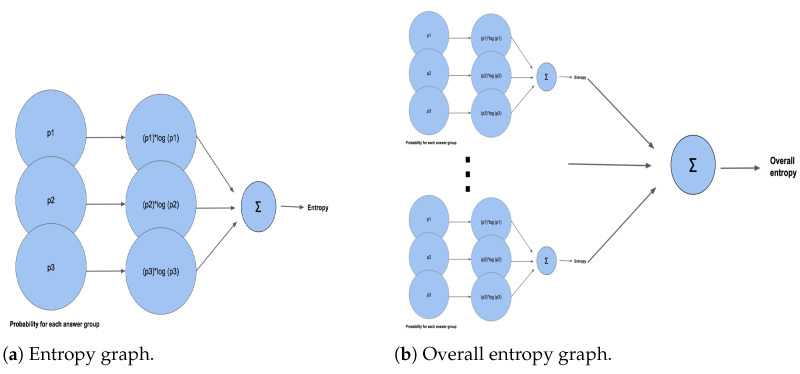
LightGBM entropy illustration.

Furthermore, we would like to discuss the entropy of the deep learning models. For each LightGBM among the 20 models, it outputs the category result with the highest probability. Let us use one model as an example. For a model that is in charge of predicting the result of User Control Question 1 (“How well does this website protect your email address?”), there are three possible answers: “Not asked for”, “Used for the internal service”, and “Shared with third parties”. For a given input, the output layer will generate a vector indicating the probabilities for the three answer groups. If the prediction result for an input privacy URL is [0.1, 0.2, 0.7], it means the probability of the corresponding privacy policy not asking for the user’s email address is 0.1. Likewise, the probability of the answer being “Used for the internal service” is 0.2. If we call the three probabilities $p_{1}$, $p_{2}$, and $p_{3}$, respectively, the prediction entropy is $\sum_{i=1}^{3}p_{i}\times \log p_{i}$. We could also define the overall entropy as $\sum_{i=1}^{n}e_{i}$, where $e_{i}$ is the entropy for each testing case. Figure 11 shows how we evaluate the entropy generated by the LightGBM models.

Entropy of the deep learning models can tell us the stability of the scoring system. The optimal system should have both high accuracy and low entropy. It is an important measurement of performance for categorical models like the one employed in the PrivacyCheck^™^ tool.

## 4. Evaluation

### Comparison between Personalized Privacy Assistants

A comparative analysis between UTCID PPA and related work highlights its advantages and distinctive features. This comparative analysis uses key features for personalized privacy assistants recommended by Colnago et al. [36]. A series of semi-structured interviews consisting of 17 participants was conducted by Colnago et al. [36] to gauge user perceptions and acceptance of personal privacy assistants, aiming to discern users’ attitudes towards the benefits and challenges associated with the autonomy of a personal privacy assistant. The study extracted key features and recommendations from these interviews, providing valuable insights for the design of privacy assistants:

- **Multiple Recommendation Sources**: Potential biases were noticed in recommendations from personal privacy assistants by users, and users expressed they preferred the ability to select preferred recommendation sources to mitigate these biases.
- **Crowd-Sourced**: Participants found recommendations based on real users’ opinions and social cues to be valuable.
- **Authoritative Sources**: Users regarded recommendations from expert opinions, manufacturers, and independent organizations as trustworthy.
- **Trusted Location Filtering**: Incorporating a “trusted location” feature to filter out unnecessary notifications about devices in familiar locations was suggested to enhance user experience.
- **Setting Configuration**: Users favored a setting configuration feature that allowed them to make decisions about device interactions and revisit these decisions periodically or as preferences change.
- **Explanations of Risks and Benefits**: Participants stressed the need for clear explanations of the risks, benefits, and consequences of data collection to make informed decisions.

Table 6 presents a comparative analysis between existing personal privacy assistants and the UTCID PPA. The key features are: Multiple Recommendation Sources (Mult-RS), Crowd-Sourcing (Crd-src), Authoritative Sources (Auth-src), Trusted Location Filtering (Tru-Loc), Setting Configuration (Set-Conf), and Explanations of Risks and Benefits (Exp-RB).

Notably, while existing works may incorporate setting configuration features, the UTCID proposed solution leverages authoritative sources like the UTCID ITAP [28] and IoT Identity Ecosystem [21], while also integrating the UTCID PrivacyCheck^™^ for additional recommendations and outcome insights. Furthermore, there are no currently existing works that provide multiple recommendation sources, crowd-sourcing, or trusted location filtering, highlighting the unique advantages of our proposed UTCID PPA. Future work may explore implementing machine learning techniques to enhance configuration of settings or to integrate additional crowd-sourced recommendation systems to further enhance user experience and decision-making abilities.

## 5. Conclusions

This research introduces innovative tools and algorithms developed by the Center for Identity at the University of Texas at Austin, namely the Personal Privacy Assistant app (UTCID PPA) that leverages the ability of the UTCID IoT Identity Ecosystem and UTCID PrivacyCheck^™^ to tackle the challenges presented by the constant influx of IoT resources (devices, services, and apps) seeking connection to users’ devices. The UTCID PPA tool aims to equip users with enhanced awareness and management of their personal information through machine learning and powerful risk analysis of exposed identity attributes.

Our findings indicate that the UTCID PPA, with its comprehensive privacy risk scoring algorithm and actionable recommendations, equips users with essential tools to manage their privacy proactively. By evaluating the data sharing policies of IoT resources and the type of personal data exposed, the UTCID PPA can calculate the entropy of privacy incident consequences, thus enabling users to make informed decisions. The integration of machine learning to analyze privacy policies via UTCID PrivacyCheck^™^ and the personalized IoT Identity Ecosystem offers users detailed insights and control over their personal data.

Compared to other personalized privacy assistants, the UTCID PPA offers unprecedented capabilities in explaining risks and benefits to the user and providing recommendations to protect their privacy. Evaluations have demonstrated its efficacy in delivering personalized privacy risk assessments for connected IoT devices, significantly contributing to user-centric privacy management.

In our opinion, the UTCID PPA sets a new standard in privacy management tools. Its ability to integrate empirical data from identity theft and fraud cases with advanced risk assessment algorithms enhances the accuracy of risk assessments and provides users with a clear understanding of the potential consequences of their interactions with IoT resources. The proactive nature of the UTCID PPA, which alerts users to privacy risks before they occur, represents a significant advancement in the field.

Looking ahead, ongoing research and development efforts should focus on refining these tools to adapt to the evolving privacy landscape. Enhancing user interface design for better accessibility and expanding the database of empirical identity theft cases can further improve the effectiveness of the UTCID PPA. Collaboration with international regulatory bodies can ensure the tool remains compliant with global privacy standards.

In conclusion, the UTCID PPA, with its advanced features and user-centric approach, is a significant contribution to privacy management. It empowers users to make informed decisions, enhancing their control over personal data in an increasingly interconnected world. Continued innovation and collaboration are crucial to maintaining this tool’s relevance and effectiveness in safeguarding user privacy in the digital age.

## Figures and Tables

**Table 1:** Summary of the relevant studies for privacy policies and IoT devices.

Category	Advantages	Gap
Devices and Privacy [2,3,4,5,6]	Concentrates on identifying actual data transmissions from mobile apps to third parties or instances of mobile apps accessing data.	Lacks provisions for user’s real-time preventive measures. Program analysis, which necessitates expert involvement, is performed offline, time-consuming, and costly. Detection of actual data transmission permits only reactive responses. Repercussions of transmitting personal data to third parties are not identified.
Privacy Risk Assessment [7,8,9,10,11,12,13,14]	Analysis of privacy policies to evaluate compliance with governance and regulatory standards.	Does not address privacy risks posed by mobile apps. Omits assessment of direct privacy risks to users.
Privacy Assistant [15,16,17,18]	Offers privacy setting recommendations to users tailored to their preferences.	Does not recommend settings based on the user’s privacy risk. Fails to explain why one identity attribute is more significant than another.

**Table 2:** Examples of identity attributes for IoT devices.

PortNumbers	PowerFrequency	PowerUsage
ProcessorType	Reputation	SerialNo
ApplicationType	BusType	Cache
CircuitDesign	Color	CookieWipe

**Table 3:** A list of mathematical symbols used in this section.

Symbol	Meaning
*V*	The set of identity attributes in the UTCID IoT Identity Ecosystem.
P(A)	The probability of exposure for identity attribute *A*.
L(A)	The liability value for identity attribute *A*.
Anc(A)	The set of ancestors for identity attribute *A*.
Des(A)	The set of descendants for identity attribute *A*.
$P^{\prime }{\left(A\right)}_{A_{k}}$	The probability that identity attribute *A* gets exposed on its own after its ancestor $A_{k}$ is exposed.
AC(A)	The *Accessibility* for identity attribute *A*.
PE(A)	The *Post Effect* for identity attribute *A*.
Exp(A)	The expected loss for identity attribute *A*.
$score_{risk}\left(A\right)$	The privacy risk score for identity attribute *A*.
Score(S)	The privacy risk score for IoT device *S*.

**Table 4:** Comparison of the target dataset and popular open-source apps.

App	Score (%)
Wiki	43.63
Firefox Focus	47.99
Kodi	48.79
QsmFurthermore	54.51
DuckDuckGo	67.39
OpenVPN	68.92
Signal Private Messenger	69.32
Ted	71.82
Blockchain Wallet	73.67
Telegram	73.99

**Table 5:** UTCID PrivacyCheck^™^ questions and scoring method [33].

	User Control	Scores: 100% (Green)	Scores: 50% (Yellow)	Scores: 0% (Red)
1	How well does this website protect your email address?	Not asked for	Used for the intended service	Shared w/ third parties
2	How well does this website protect your credit card information and address?	Not asked for	Used for the intended service	Shared w/ third parties
3	How well does this website handle your Social Security number?	Not asked for	Used for the intended service	Shared w/ third parties
4	Does this website use or share your location?	PII not used for marketing	PII used for marketing	PII shared for marketing
5	Does this website track or share your location?	Not tracked	Used for the intended service	Shared w/ third parties
6	Does this website collect PII from children under 13?	Not collected	Not mentioned	Collected
7	Does this website share your information with law enforcement?	PII not recorded	Legal docs required	Legal docs not required
8	Does this website notify or allow you to opt out after changing their privacy policy?	Posted w/ opt-out option	Posted w/o opt-out option	Not posted
9	Does this website allow you to edit or delete your information from its record?	Edit/delete	Edit only	No edit/delete
10	Does this website collect or share aggregated data related to your identity or behavior?	Not aggregated	Aggregated w/o PII	Aggregated w/ PII
	**GDPR**	**Scores: 100% (Green)**		**Scores: 0% (Red)**
1	Does this website share the user’s information with other websites only upon user consent?	Yes		No/Unanswered
2	Does this website disclose where the company is based/user’s PII will be processed and transferred?	Yes		No/Unanswered
3	Does this website support the right to be forgotten?	Yes		No/Unanswered
4	If they retain PII for legal purposes after the user’s request to be forgotten, will they inform the user?	Yes		No/Unanswered
5	Does this website allow the user the ability to reject their usage of user’s PII?	Yes		No/Unanswered
6	Does this website restrict the use of PII of children under the age of 16?	Yes		No/Unanswered
7	Does this website advise the user that their data are encrypted even while at rest?	Yes		No/Unanswered
8	Does this website ask for the user’s informed consent to perform data processing?	Yes		No/Unanswered
9	Does this website implement all of the principles of data protection by design and by default?	Yes		No/Unanswered
10	Does this website notify the user of security breaches without undo delay?	Yes		No/Unanswered

**Table 6:** Existing works and features of personalized privacy assistants.

Work	Mult-RS	Crd-Src	Auth-Src	Tru-Loc	Set-Conf	Exp-RB
UTCID PPA	Yes		Yes			Yes
Das et al. [15]					Yes	
Feng et al. [16]					Yes	
Ayci et al. [17]					Yes	

## Data Availability

No new data were created or analyzed in this study. Data sharing is not applicable to this article.

## Abbreviations

The following abbreviations are used in this manuscript:

DNS	Domain Name System
IoT	Internet of Things
IRR	IoT Resource Registry
ITAP	Identity Threat Assessment and Prediction
NLP	Natural Language Processing
PII	Personally Identifiable Information
PPA	Personal Privacy Assistant
UTCID	University of Texas at Austin Center for Identity
